# Associations of adverse childhood experiences and social vulnerability with a diagnosis of cognitive impairment or dementia

**DOI:** 10.1002/alz70858_103478

**Published:** 2025-12-24

**Authors:** Judith Godin, Selena P Maxwell, Jasmine Mah, Maia von Maltzahn, Lindsay MK Wallace, Shanna Claire Trenaman, Olga Theou, Kenneth Rockwood, Melissa K. Andrew

**Affiliations:** ^1^ Nova Scotia Health, Halifax, NS, Canada; ^2^ Dalhousie University, Halifax, NS, Canada

## Abstract

**Background:**

Adverse childhood experiences and social vulnerability in adulthood are associated with poorer health outcomes including cognitive impairment and dementia. One proposed mechanism linking adverse childhood experiences with later development of dementia is the association with other known social risk factors such as lower education. Here, we consider the association of social vulnerability, a comprehensive measure of social risk factors, and adverse childhood experiences with a clinical diagnosis of cognitive impairment or dementia.

**Method:**

We analyzed data from 949 participants in the COMPASS‐ND study of neurodegenerative disorders. Social vulnerability was measured using a deficit accumulation approach resulting in a 49‐item social vulnerability index (SVI). Early childhood experience was assessed using the Adverse Childhood Experiences Questionnaire and participants were grouped into 0, 1‐2, and 3 or more adverse childhood experiences. Multinomial logistic regression was used to examine associations between adverse childhood experiences and social vulnerability with clinical diagnosis (i.e., dementia, mild cognitive impairment (MCI), and subjective cognitive impairment (SCI) compared to cognitively unimpaired). Models included adverse childhood experiences, the SVI, and were adjusted for age, education, and an 81‐item frailty index. Analyses were conducted separately for each sex with clinical diagnosis as the outcome.

**Results:**

Ages ranged from 42‐90 years (M=72, SD=7.3) and 48.3% were female. For females, there was no statistically significant association between adverse childhood experience and diagnoses. Every 0.1 difference in the SVI was associated with higher odds of MCI (OR = 1.41, 95%CI=1.11‐1.80) and SCI (OR=1.53, 95%CI=1.18‐1.98) compared to cognitively unimpaired females. For males, neither adverse childhood events nor social vulnerability had a statistically significant association with diagnoses.

**Conclusion:**

Being more socially vulnerable as an adult is associated with higher odds of experiencing mild or subjective cognitive impairment in females. As this is a cross‐sectional study, we do not know the direction of the association and it is likely bi‐directional to some degree. Strategies for reducing social vulnerability may also provide bi‐directional benefits in improving the lives of females who have cognitive impairment and providing some protection against cognitive decline.

